# “Inflammaging” as a Druggable Target: A Senescence-Associated Secretory Phenotype—Centered View of Type 2 Diabetes

**DOI:** 10.1155/2016/1810327

**Published:** 2016-06-01

**Authors:** Francesco Prattichizzo, Valeria De Nigris, Lucia La Sala, Antonio Domenico Procopio, Fabiola Olivieri, Antonio Ceriello

**Affiliations:** ^1^August Pi i Sunyer Biomedical Research Institute (IDIBAPS) and Centre of Biomedical Investigation on Diabetes and Associated Metabolic Disorders Network (CIBERDEM), 08036 Barcelona, Spain; ^2^Department of Clinical and Molecular Sciences, DISCLIMO, Università Politecnica delle Marche, 60126 Ancona, Italy; ^3^IRCCS Multimedica Group, 60126 Sesto San Giovanni, Italy; ^4^Center of Clinical Pathology and Innovative Therapy, Italian National Research Center on Aging (INRCA-IRCCS), 60121 Ancona, Italy

## Abstract

Aging is a complex phenomenon driven by a variety of molecular alterations. A relevant feature of aging is chronic low-grade inflammation, termed “inflammaging.” In type 2 diabetes mellitus (T2DM), many elements of aging appear earlier or are overrepresented, including consistent inflammaging. T2DM patients have an increased death rate, associated with an incremented inflammatory score. The source of this inflammation is debated. Recently, the senescence-associated secretory phenotype (SASP) has been proposed as the main origin of inflammaging in both aging and T2DM. Different pathogenic mechanisms linked to T2DM progression and complications development have been linked to senescence and SASP, that is, oxidative stress and endoplasmic reticulum (ER) stress. Here we review the latest data connecting oxidative and ER stress with the SASP in the context of aging and T2DM, with emphasis on endothelial cells (ECs) and endothelial dysfunction. Moreover, since current medical practice is insufficient to completely suppress the increased death rate of diabetic patients, we propose a SASP-centered view of T2DM as a futuristic therapeutic option, possibly opening new prospects by moving the attention from one-organ studies of diabetes complications to a wider targeting of the aging process.

## 1. Introduction

Aging is an intricate process that results from a combination of environmental, genetic, epigenetic, and stochastic factors [1]. A chronic proinflammatory status is a pervasive feature of aging. This chronic, low-grade, systemic inflammation occurring in the absence of overt infection (sterile inflammation) has been defined as “inflammaging” and represents a significant risk factor for morbidity and mortality in the elderly [2]. There is growing epidemiological evidence that a state of mild inflammation is associated with and predicts several age-related diseases (ARDs), including type 2 diabetes mellitus (T2DM) and its complications (e.g., cardiac death) [3–5]. According to the tenets of inflammaging, both the aging process* per se* and ARD development are fostered by an inescapable age-dependent proinflammatory drift [2]. The etiology of inflammaging and its contribution to ARDs development are the subject of intense research work.

The life expectancy of T2DM patients is about 6 years shorter than that of nondiabetic individuals of similar age [6]. Analysis of the factorial structure changes that take place during aging has disclosed that healthy aging involves a decrease in complexity and a concomitant increase in variability and inflammation. Interestingly, a decrease in complexity seems to arise earlier in diabetes patients [7], suggesting that under some respects diabetes may provide an interesting model of “accelerated aging.” Diabetes patients may thus experience an accelerated aging process that increases the risk of developing frailty and morbidity and of an earlier death [8]. Although the role of inflammation in the pathogenesis of T2DM and its complications is well established [5, 9], the underlying molecular mechanisms are debated. Together with immunological factors, cellular senescence and the senescence-associated secretory phenotype (SASP) are currently held to be the largest contributors to inflammaging [1]; however, a key role of senescence in patients with the most common ARDs (e.g., diabetes) has yet to be conclusively demonstrated. Significantly, at least two major molecular changes responsible for diabetes complications and also associated with physiological aging and T2DM, that is, oxidative stress and endoplasmic reticulum (ER) stress [10, 11], have recently been related to senescence acquisition and/or SASP modulation [12, 13]. These findings suggest that the SASP can contribute to the endothelial dysfunction characterizing aging as well as T2DM.

Here we review the latest data connecting oxidative and ER stress with the SASP in the context of aging and T2DM, with emphasis on endothelial cells (ECs) and endothelial dysfunction. Moreover, since current lifestyle interventions and medications are unable to reduce the mortality of diabetic patients from cardiovascular disease, we also outline a gerontological, SASP-centered view of the vascular complications of diabetes that could provide a broader range of therapeutic options.

## 2. Endothelial Senescence: A Central Player in Aging and Diabetes Complications

Aging is accompanied by a progressive endothelial dysfunction that affects both diabetes patients and healthy individuals [14]. This widely accepted notion suggests that vascular aging—where vessel walls undergo profound remodeling including lumen enlargement, intimal and medial thickening, and increased vascular stiffness combined with loss of endothelial barrier integrity and function—is a key factor in organismal aging and in the development of diabetes complications [15–17]. These changes have recently been related to the onset of a proinflammatory program in ECs and vessel wall cells [18]. Inflammation is a nonspecific stereotypical response elicited by a variety of stimuli, many of which have been implicated in aging and T2DM [1, 2]. We have designated the secretory phenotype acquired by ECs “endo-SASP” [19]. The rate of senescent cell accumulation in the body depends on the balance between pro- and antisenescence stimuli [20]. However, the sheer number (about 60 trillion) of ECs and the area covered by them (about 4000–7000 m^2^) suggest that SASP acquisition by these cells must exert a strong effect at the systemic level [21]. In particular, the buildup of senescent ECs is held to foster inflammaging. Indeed,* ex vivo* endothelium from relatively healthy aged and T2DM individuals shows chronic nuclear factor-*κ*B (NF-*κ*B) expression and activation [22, 23]. Moreover, blockade of NF-*κ*B-mediated inflammatory responses in the endothelium prolongs lifespan by preventing insulin resistance [24]. These findings clearly point at the endothelium as a key organ in the pathophysiology of aging and in diabetes complications.

## 3. Senescence, SASP, and Inflammaging

Cellular senescence is associated with the acquisition of the SASP, which is characterized by the activation of a proinflammatory transcriptional program and is held to drive ARD development through chronic secretion of a variety of factors [1, 25, 26]. Interestingly, the main pathways involved in cellular senescence regulation, such as the NF-*κ*B, the mechanistic target of rapamycin (mTOR), and the interleukin-1/NLR family pyrin domain containing 3 (IL-1/NLRP3) inflammasome pathways, are master modulators of the aging rate [27–29]. In animal models, the number of senescent cells in specific organs predicts lifespan [30]. Notably, in animal models removal of senescent cells is sufficient to prolong lifespan and healthspan, as described in mice models of accelerated aging (INK-ATTAC mice) and in models of physiological aging [31, 32]. Evidence that the number of senescent dermal fibroblasts correlates with ARD incidence has also been provided in humans [33].

A variety of exogenous and endogenous stimuli, including replicative exhaustion, oncogene activation, oncosuppressor loss, DNA damage, irradiation, viral infection, oxidative stress, and hyperglycemia, are capable of inducing a state of permanent cell cycle arrest accompanied by a sustained proinflammatory program. In proliferating human cells, progressive telomere erosion eventually leads to exposure of an uncapped free double-stranded chromosome end, triggering a permanent DNA damage response (DDR). The damage sensor ataxia telangiectasia mutated (ATM) is subsequently recruited to the uncapped telomeres, fostering the stabilization of tumor suppressor protein 53 (p53) and upregulating its target p21. In turn, p21 prevents inactivation of retinoblastoma tumor suppressor protein (RB), thus inhibiting entry into the S phase of the cell cycle [26, 34]. Other DNA-damaging stressors, such as ultraviolet radiation, chemotherapeutic drugs, hyperglycemia, and oncogenic Ras overexpression, also engage the ATM-p53-p21 axis [35–37]. This senescence pathway can also be engaged by loss of the tumor suppressor phosphatase and tensin homolog (PTEN) and overexpression of the S-phase E2F transcription factor 3 (E2F3) [26, 34]. p16^Ink4a^ is a further barrier to proliferation, preventing cyclin-dependent kinase-4/6- (CDK4-/CDK6-) mediated inactivation of RB, thus blocking cell cycle progression [26, 34]. This mechanism can act either alone or in combination with the p53-p21 pathway, depending on stressor or cell type. Therefore, different prosenescence stimuli may engage different pathways [26, 34], leading to partially different gene expression patterns [38]. However, a large panel of secreted factors is characteristic of the senescence state induced by all stimuli in all cell types. Indeed, the SASP involves release of hundreds of molecules [35–38], of which interleukin- (IL-) 1 *α*/*β*, IL-6, IL-8, transforming growth factor- (TGF-) *β*, and tumor necrosis factor- (TNF-) *α* are the most common and best characterized [35, 36, 38]. Some of these cytokines can induce or reinforce the senescent phenotype by acting in an autocrine and paracrine manner, spreading senescence via a “bystander effect” [36]. In particular, IL-1*α* appears to be the main upstream regulator of the SASP, while IL-1*β* and TGF-*β* are mediators of senescence transmission, and IL-6 and IL-8 reinforce autocrine senescence [36, 39–42]. These data suggest that regardless of the “first culprit,” tissue ages “senescence by senescence.”

A number of adaptor proteins have been shown to control SASP factor secretion. The SASP is mostly induced by NF-*κ*B, the main immunological transcription factor [43]. Upstream, p38 [44], JAK [45], and other MAP kinases [46], are all involved in SASP induction and control. Interestingly, Jak2/Stat3 pathway inhibition redesigns the SASP, suppressing the secretion of certain factors and increasing others (i.e., MCP-1), indicating that selective SASP modulation could be feasible [47]. Finally, highly interesting results in terms of SASP suppression have been obtained with the mTOR inhibitor rapamycin [48, 49], which has long been known to extend lifespan and healthspan in mice [50]. mTOR controls SASP protein secretion by enhancing IL-1*α* and MAP kinase-activated protein kinase 2 (MAPKAP2) translation [48, 49].

*In vivo* measurement of senescence and the SASP is of critical importance. Number of senescence-associated heterochromatin foci (SAHF), number and phosphorylation of *γ*-H2AХ foci, levels of the heterochromatin protein HP1*γ*, P53-binding protein 1 (53ВР1), H3K9me3, and other markers have been measured both in senescent cells and nonsenescent cells “stressed” in various ways (e.g., radiation, hyperglycemia, oxidative stress, and ER stress) [51], suggesting a close interconnection among such phenomena. The expression of p16Ink4a, the end effector of cell cycle arrest, and SA-*β*-galactosidase enzyme activity are currently the most widely used surrogate markers of senescence [26], even though the possibility of false positive results has been reported for both [52, 53]. Similarly, the inflammatory components of the SASP are shared by nearly all types of inflammatory responses. Unfortunately, research efforts are still hampered by the lack of a universally accepted standard to identify senescent cells and determine their functional significance* in vivo* [26].* In vivo *senescence probably encompasses a spectrum of states ranging from low to high SASP expression, depending on its inducers (replication, oxidative stress, oncogene activation) and cell types, among other factors. The chronic senescent state characteristic of natural aging is likely to be a complex phenomenon induced by some combination of telomere attrition, (oxidative) DNA damage, ER stress, and other slowly accumulating forms of macromolecular damage [26].

Interventions directed at preventing the adverse effects associated with the SASP are being explored [25, 26]. The most promising strategies involve delaying cellular senescence [30]; SASP switch-off [48, 49]; SASP factor modulation [34, 47]; and selective removal or killing of existing senescent cells (senolytics or senotherapy) [25, 54]. Selective targeting and killing of senescent cells without damaging neighboring, healthy cells requires identifying senescence-associated markers and devising strategies to exploit them [25, 26]. High-throughput “omics” technologies (i.e., genomics, metabolomics, metagenomics, and transcriptomics) are being applied to discover such markers [55]. Highly promising results are coming from work on SASP suppressor and senolytic agents [26, 48–54].

## 4. SASP, Inflammaging, and Type 2 Diabetes

Several ARDs, including T2DM and cardiovascular diseases, share a chronic, low-grade inflammatory state [1, 2]. All lines of evidence point at low-grade inflammation as a key T2DM therapeutic target [9]. According to a recent hypothesis, the buildup of cells expressing the SASP may promote the development of both diabetes and its vascular complications [26, 56]. SASP genes (i.e., IL-1*α*, IL-1*β*, IL-6, and TNF-*α*) are chronically activated in cells and tissues from diabetes patients [23]. The role of senescence in the development of T2DM and related vascular complications and whether it predates or follows the onset of low-grade inflammation and vascular complications are the subject of intense research work. An outstanding question is whether the glucose-related metabolic perturbations of T2DM promote telomere attrition and increase DNA oxidative damage or accelerated cellular senescence in multiple cell types, including *β* cells, adipose tissue, and endothelium, is a pathogenic mechanism involved in T2DM development and progression [56]. Epigenetic modifications leading to chronic inflammation have been described in ECs and immune cells of diabetes patients also in the absence of replicative senescence biomarkers [23, 57, 58]. However, the majority of the inflammatory mediators involved in the vascular complications of diabetes, which are induced* in vitro* in ECs and immune cells by hyperglycemia, are the SASP-modulating and SASP-released molecules, like NF-*κ*B, IL-1, IL-6, TNF-*α*, and vascular cell adhesion molecule-1 (VCAM-1) [23, 58], suggesting that the SASP has a causal role in maintaining the chronic, systemic inflammation that is associated with diabetes. A comparative analysis of gene expression in replicative and hyperglycemia-induced senescence could shed some light on the question. Moreover, circulating cytokine concentrations can be increased by hyperglycemia, also in an acute manner, through an oxidative mechanism, whose effect is more marked in subjects with impaired glucose tolerance [59]. High-glycemic index carbohydrate increases NF-*κ*B activation in peripheral blood mononuclear cells (PBMCs) and this proinflammatory effect could be partially reversed by prandial basal insulin treatment [60, 61]. Hyperglycemia thus promotes the acquisition of a proinflammatory cellular phenotype that may be defined as a diabetes- or hyperglycemia-associated secretory phenotype (DASP or HASP). The high-level oxidative stress induced by hyperglycemia and other imbalances associated with T2DM (i.e., altered hormonal status and lipid metabolism, epigenetic alterations, and low-grade inflammation) have the potential to foster premature senescence [56, 60]. Senescent cell accumulation can therefore contribute to spread senescence at the systemic level, further fueling low-grade inflammation and promoting T2DM progression [56, 57]. Consequently, senescent cells might be part of a pathogenic loop both as a cause and a consequence of metabolic changes and tissue damage in diabetes [56, 62]. This hypothesis is partially supported by the observation that leukocyte telomere length (LTL), a surrogate marker of senescence, has a nonlinear association with incident diabetes, indicating that it could serve to predict T2DM development [63]. Moreover, a clear, inverse age-dependent association between LTL and insulin resistance has been documented [64]. LTL is significantly shorter in T2DM patients compared with controls over a wide age range and is significantly associated with the presence and number of diabetes complications [65]. Finally, a strong association has been highlighted between telomere length and the presence of some diabetes complications and general arterial aging [66, 67].

Evidence of significant* in situ* senescence of cells with a functional SASP in adipose tissue (mainly ECs and adipocyte progenitors) from obese subjects has been linked to T2DM development [37, 68]. As regards pancreatic *β*-cells, even though aging in itself does not appear to be critical in inducing tissue dysfunction [69, 70], increased *β*-cell senescence during aging and a limited regenerative potential have been described in senescent mouse pancreas [71]. After the initial* in vitro* demonstration that exposure to high glucose levels induces EC senescence [72],* in vivo* evidence of increased senescence has been provided in atherosclerotic plaques and kidney tissue of diabetes patients, suggesting the relevance of this phenomenon to T2DM vascular complications [73, 74]. Moreover, mounting evidence suggests an important role for the inflammasome platform in both T2DM and atherosclerosis [75, 76]. The NOD-like receptor- (NLR-) caspase 1-IL-1*β* cascade can be activated by endogenous metabolism or injury-derived byproducts called damage-associated pattern molecules (DAMPs), resulting in chronic secretion of inflammatory cytokines [77]. Strikingly, the inflammasome controls transmission of the SASP senescence signal [39]. In turn, it is widely accepted that oxidative stress and macromolecular damage play a major role in controlling the inflammasome platform [75, 78, 79], mainly through the Trx/TXNIP complex, linking redox status, ER stress, and inflammation. NLRs likely respond to some generic cellular stress signals induced by the multiple molecules that trigger its activation. In addition to NLR activation, toll-like receptor (TLR) activation has also been proposed to be closely involved in T2DM and its complications, supporting a role for innate immunity and probably microbiota in the diabetic inflammatory milieu [80, 81].

Clinical trials involving IL-1 blockade in T2DM patients have provided promising results. Anakinra (a recombinant human IL-1 receptor antagonist) improved glycemia and *β* cell secretory function and reduced the levels of systemic inflammation markers [82]. Canakinumab, a human monoclonal antibody that neutralizes IL-1*β*, also significantly reduced inflammation and glycated hemoglobin in diabetes patients [83, 84]. Large clinical trials are exploring the potential of IL-1 antagonism to prevent cardiovascular and other diabetes complications [85]. Anticytokine agents are commonly used to treat autoimmune diseases, which are characterized by chronic inflammation, endothelial dysfunction, accelerated aging, and a heavy burden of senescent cells [86, 87]. The three licensed anti-TNF-*α* biologics have shown effectiveness in improving endothelial function; similar results have been obtained with anti-IL-6 treatment [88]. However, clinical trials that block these cytokines in T2DM patients are yet to be set up.

The pathophysiology of T2DM complications is characterized by increased oxidative stress, nonenzymatic glycation, and PKC overactivation as the main molecular changes involving micro- and macrovascular compartments [89]. However, targeting these changes with innovative therapies has had limited success in slowing down disease progression and the development of complications [90], indicating that not all the imbalances experienced by diabetes patients can be addressed by one-target treatments. The pathways involved in the vascular complications of T2DM are complex, interlinked, and self-perpetuating. It is unlikely for a single druggable pathway to prevent their onset, probably also because the intricate connections among the mechanisms giving rise to diabetes complications create redundancy [90]. Moreover, current diabetes treatments are unable to halt the development of vascular complications, especially in patients with long-standing disease [91]. Therefore, if a large role for senescence and the SASP in T2DM is confirmed, new therapies aimed to remove senescent cells or counter the noxious effect of the SASP might be suitable alternatives to slow down T2DM progression and delay complication onset. For instance, rapamycin, the main SASP-suppressing agent, reduces renal hypertrophy in diabetic mice and slows the progression of diabetic kidney disease in rats, even without reducing blood glucose [92]. Prevention of age-related macular degeneration-like retinopathy by rapamycin has also been reported [93]. A theoretical model where mTOR is viewed as the central mediator of insulin resistance and diabetes complications has been proposed [94]. A putative antisenescence* in vivo* role [95] and an anti-SASP* in vitro *action [96] have been proposed for metformin, currently the most effective antidiabetes agent in terms of prevention of its vascular complications [91]. Finally, an agent interfering with the glycolysis pathway (2-deoxyglucose) has been shown to selectively affect senescent cells by exerting an antisurvival action, thus attenuating inflammation [97].

## 5. Oxidative Stress-Induced Senescence and Redox Control of the SASP 

A large number of studies have documented the ability of antioxidant compounds to delay senescence onset* in vitro*. In ECs this has clearly been demonstrated for coenzyme Q10 [98], N-acetyl cysteine [99], and a stable vitamin C analog [100]. However, clinical studies have failed to confirm the benefit of antioxidants and vitamins in cardiovascular disease prevention and management [101]. A number of reasons may account for this: (i) nonoptimal pharmacokinetic profiles, preventing antioxidants from reaching the endothelium or from maintaining a stable concentration of the agent over time; (ii) overestimation of the* in vitro* effect of antioxidants, since experiments are usually performed under normal oxygen tension, whereas oxygen levels in the body are considerably lower, especially in deep tissue layers; (iii) possible downregulation of cellular antioxidant defenses (i.e., superoxide dismutase, catalase) following prolonged exposure to the antioxidant (pharmacodynamic tolerance); and (iv) the possible sensitivity of the endothelium, which is continuously exposed to various stressors, to reactive oxygen species (ROS) dosage and the existence of a putative beneficial effect of low ROS levels* in vivo* (hormesis) [102, 103]. Notably, recent experimental evidence strongly suggests that low ROS levels extend lifespan in different model organisms [102]. Therefore, a more useful definition of oxidative stress may be “a state where oxidative force exceeds the antioxidant systems due to loss of the balance between them” [104]. More promising results have been obtained in T2DM patients and animal models. Endothelial progenitor cells (EPCs) from diabetic donors undergo oxidative stress-induced premature senescence [105]. EC senescence, with p53 and p16 upregulation in endothelium, also occurs in the arteries of rats with T2DM [106]. Importantly, these animals display evidence of vascular dysfunction, and antioxidant treatment prevents endothelial senescence, ameliorating endothelial dysfunction. Similarly, diabetic patients treated with antioxidant compounds show improved endothelial function and inflammatory profiles [107]. However, improvement of long-term outcomes in T2DM patients has not been detected [108]. Enhancing cellular antioxidant defenses has been proposed as an alternative to antioxidant administration to restore EC physiological redox status and prevent T2DM- or aging-induced cardiovascular disease by reducing hyperglycemia- and/or aging-related damage [108]. For example, caloric restriction (CR), which has well-established lifespan-promoting action, exerts persistent antioxidant and anti-inflammatory cellular effects, preserving a youthful phenotype in several cell types including ECs. Among nutrient-sensitive factors, sirtuins, a family of NAD^+^-dependent deacetylases with epigenetic modulation activity, can prevent vascular endothelial replicative senescence by increasing antioxidant defense [109, 110]. CR mimetics like resveratrol and other synthetic sirtuin activators confer broad health benefits including preservation of endothelial function and attenuation of low-grade inflammation, especially in obesity and T2DM models [109–111].

Besides senescence, the SASP is also controlled by the cellular redox status [12]. IL-1*α* is a key senescence-associated proinflammatory cytokine acting as a critical upstream regulator of the SASP. Senescence-associated shifts in steady-state H_2_O_2_ and intracellular Ca^2+^ levels induce increased IL-1*α* expression and processing. Increased intracellular Ca^2+^ promotes calpain activation and the proteolytic cleavage of IL-1*α* [12]. Antioxidants and low oxygen tension prevented senescence-associated IL-1*α* expression and reduced the expression of SASP components IL-6 and IL-8. Similarly, Ca^2+^ chelation or calpain inhibition impaired senescence-associated processing of IL-1*α* and its ability to induce downstream cytokine expression [12].

## 6. Endoplasmic Reticulum Stress, Diabetes, and the SASP

Over the past decade, ER stress has emerged as a new factor in the pathogenesis of diabetes and its complications. A considerable number of recent studies have highlighted its role in the onset of insulin resistance, hyperglycemia, and endothelial dysfunction [112].

Pathological conditions involving altered ER homeostasis, such as overexpression of misfolded proteins, low ER chaperone levels and Ca^2+^ content, ER phospholipid depletion and cholesterol accumulation, and changes in the redox status that occur in diabetes, induce a state of ER stress that leads to activation of Unfolded Protein Response (UPR) [112, 113]. ER stress is an imbalance between the proper folding and the secretory capacity of the ER that results in accumulation of misfolded proteins. Loss of ER homeostasis activates the ER stress response, a crucial adaptive mechanism in secretory cells, which serves to dynamically expand ER size and capacity to meet the functional demand placed on the exocytosis pathway [112]. UPR markers are overexpressed in the liver and adipose tissue of diabetic rodents [113] as well as in* ex vivo* samples from T2DM patients [114].

Chronic ER stress and UPR activation may also result in ROS buildup, which induces a state of oxidative stress [115]. ROS overproduction and the development of oxidative stress can thus be viewed both as a cause and a consequence of ER stress. ROS accumulation is due to UPR-stimulated upregulation of chaperone proteins involved in disulfide bond formation in the ER lumen. Chaperones use oxidation/reduction reactions, with molecular oxygen as the final electron recipient. The reduced molecular oxygen accumulates during increased protein folding due to UPR activation, resulting in cell toxicity [116].

Diabetes or hyperglycemia induce ER stress in many organs [112–116], including endothelium [117]. Many biochemical and molecular imbalances found in T2DM subjects are ER stress inducers. Altered nutrient availability, free fatty acids, cytokines, perturbations in calcium transients, oxidative stress, and hypoxia are all capable of triggering accumulation of unfolded ER proteins [118].

Interestingly, recent papers strongly link ER stress with SASP. In particular, a negative feedback loop mediated by macro H2A1.1 limits ER stress during senescence by suppressing SASP gene expression [13]. ER stress is a feature of senescence triggered by the expression of some secreted SASP molecules [119, 120]. The formation of correct disulfide bonds is critical for many SASP components. For instance, CXC-type cytokines such as IL-8 require the formation of two disulfide bonds for folding and function [121]. Disulfide bond rearrangement in the ER is catalyzed by protein disulfide isomerases (PDIs), which are critical for the folding of disulfide-containing secreted factors [122]. Increased PDI activity due to unfolded proteins can then increase ROS levels, chiefly through NADPH oxidase 4 (NOX4) upregulation [122]. In turn, NOX4 is a prooxidant enzyme capable of triggering senescence [123].

Canonical UPR pathways try to mitigate ER stress through several mechanisms that include increased ER chaperone synthesis, inhibition of translation, and regulated IRE1-dependent decay (RIDD) of mRNA [124]. The increased ROS levels caused by ER stress induce DNA damage, activating a DDR involving ATM [125]. Active ATM elicits macro H2A1 removal from SASP genes, keeping ER stress in check, a process called reactive oxygen and ATM-mediated macro H2A1 mobilization (ROAMM) pathway [13]. The ROAMM pathway explains why ATM inhibition leads to steep increases in SASP gene expression and ER stress. In the absence of ATM-mediated negative feedback from ER stress, the SASP positive feedback loop runs unopposed, inducing a dramatic increase in both SASP expression and ER stress [13].

## 7. Conclusions and Future Directions

Epidemiological, biochemical, and molecular lines of evidence suggest that aging and T2DM share a number of important features that include oxidative stress, ER stress, endothelial dysfunction, and low-grade inflammation. SASP is modulated by or modulates all these mechanisms and may be a druggable element of connection. Several lines of research are trying to harness some molecular features of aging in the context of T2DM, like sirtuin family activators, mTOR inhibitors, and ER stress modulators. The route to clinical translation requires further, decisive insights into the role of senescence/SASP in T2DM. For instance, crossing T2DM mice models with the INK-ATTAC model (characterized by automated clearance of senescent cells) would provide definitive information. T2DM could be the ideal model to test senolytics and other SASP-modifying drugs. T2DM is very likely to benefit from a shift from single pathway blockade to broader targeting of the senescence process. A SASP-centered or simply gerontological view of T2DM could open new prospects by moving the focus from single organ studies of diabetes complications to their long-term consequences, possibly improving the condition of those patients whose higher risk of death is not mitigated by current disease management approaches.
